# Vasoactive intestinal peptide inhibits the production of Salmonella-induced inflammatory cytokines by human monocytes

**DOI:** 10.1186/cc14030

**Published:** 2014-12-03

**Authors:** B Askar, P Barrow, N Foster

**Affiliations:** 1 Animal Infection and Immunity, School of Veterinary Medicine and Science, University of Nottingham, UK

## Introduction

Systemic salmonella infection is a frequent cause of Gram-negative sepsis. Bacterial lipopolysaccharide in blood triggers immune response by monocytes, which results in overwhelming production of proinflammatory cytokines and pathology in peripheral organs such as the liver, heart and lungs. The mortality rate due to sepsis remains high even after chemotherapeutic clearance of pathogens, due to sustained production of inflammatory mediators. Therefore, anti-inflammatory therapy as an adjunct to antibiotics could reduce the mortality from sepsis.

## Methods

To date, several studies have evaluated the role of vasoactive intestinal peptide (VIP) as a therapeutic agent in sepsis both *in vivo *and *in vitro *since it possesses several desirable biological properties. The peptide, acting via VIP cell receptors, mediates effects by altering production and secretion of inflammatory mediators such as cytokines. VIP has been shown to decrease production of proinflammatory cytokines, ameliorate histopathological changes and inhibit mortality in mice rendered septic by LPS administration. Nothing is known about the effect of VIP on production of proinflammatory cytokines in human monocytes infected by virulent Salmonella.

The aim of the current study, therefore, was to investigate the effect of vasoactive intestinal peptide on the production of inflammatory cytokines in human monocytes exposed to *S. Typhimurium *4/74.

## Results

Our finding demonstrates that freshly isolated human monocytes produce proinflammatory cytokines such as TNFα, IL-6, IL-1β and also anti-inflammatory cytokines such as IL-4 and IL-10 following bacterial challenge.

## Conclusion

However, co-culture of infected monocytes with VIP (10-7 M) significantly reduced (*P *< 0.05) production of TNFα, IL-6, IL-1β but significantly increased (*P *< 0.05) concomitant production of anti-inflammatory IL-10.

